# Geographic Variations on the Safety and Efficacy of the Supreme Biodegradable Polymer DES: Results From PIONEER III

**DOI:** 10.1016/j.jscai.2022.100515

**Published:** 2022-11-25

**Authors:** Philippe Garot, Martin B. Leon, Shigeru Saito, Andreas Baumbach, Dean J. Kereiakes, Stephan Windecker, Cody Pietras, Ovidiu Dressler, M. Ozgu Issever, Michael Curtis, Barry D. Bertolet, James P. Zidar, Pieter C. Smits, Victor Alfonso Jiménez Diaz, Brent McLaurin, Angel R. Cequier, Sjoerd H. Hofma, Nabil Dib, Atsuo Namiki, Akihiko Takahashi, Tsunekazu Kakuta, Atsushi Hirohata, Alexandra J. Lansky

**Affiliations:** a Institut Cardiovasculaire Paris-Sud, Hôpital Jacques Cartier, Ramsay-Santé, Massy, France; b NewYork-Presbyterian/Columbia University Irving Medical Center, New York, New York; c Cardiovascular Research Foundation, New York, New York; d Shonan Kamakura General Hospital, Kamakura, Japan; e Division of Cardiology, Yale School of Medicine, New Haven, Connecticut; f Centre for Cardiovascular Medicine and Devices, William Harvey Research Institute, Queen Mary University of London and Barts Heart Centre, London, United Kingdom; g Christ Hospital Heart and Vascular Institute, Cincinnati, Ohio; h Department of Cardiology, Bern University Hospital, Inselspital, University of Bern, Switzerland; i University of Calgary, Alberta, Canada; j Cardiology Associates of North Mississippi, Tupelo, Mississippi; k North Carolina Heart and Vascular, University of North Carolina, Raleigh, North Carolina; l Maasstad Ziekenhuis, Rotterdam, the Netherlands; m Hospital Álvaro Cunqueiro, Vigo, Spain; n AnMed Health Medical Center, Anderson, South Carolina; o Bellvitge University Hospital, University of Barcelona, IDIBELL, Barcelona, Spain; p Medisch Centrum Leeuwarden, Hartcentrum Friesland, Leeuwarden, the Netherlands; q Mercy Gilbert Medical Center, Gilbert, Arizona; r Department of Cardiology, JOHAS Kanto-Rosai Hospital, Kanagwa, Japan; s Department of Cardiology, Sakurakai Takahashi Hospital, Hyogo Japan; t Department of Cardiology, Tsuchiura Kyodo General Hospital, Ibaraki, Japan; u Department of Cardiology, The Sakakibara Heart Institute of Okayama, Okayama, Japan

**Keywords:** drug-eluting stents, geography, health care, outcomes, percutaneous coronary intervention

## Abstract

**Background:**

The PIONEER III trial showed the 12-month safety and efficacy of the Supreme drug-eluting stent (DES) vs the durable polymer everolimus-eluting stent. We sought to assess whether the characteristics and clinical outcomes of the Supreme DES in PIONEER III were consistent among patients by enrollment location.

**Methods:**

This subgroup analysis of the PIONEER III trial compared the characteristics and outcomes of patients recruited from North America, Europe, and Japan and the relative differences in patient outcomes according to the site recruitment volume.

**Results:**

From October 2017 to July 2019, 1629 patients were recruited in North America (816, 50.1%), Europe (650, 39.9%), and Japan (163, 10%). Procedural success was achieved in 1556 of 1611 procedures (96.6%), with no difference by the geographic location. Target lesion failure at 12 months for combined groups was observed in 84 of 1629 patients (5.2%), with no significant geographic differences (4.7%, 6.5%, and 2.5%, respectively; *P* =.08), with similar results in the Supreme DES group alone (4.4%, 6.8%, and 3.7%, respectively, *P* =.20). Cardiac death at 12 months occurred in 0.4%, 0.2%, and 0.0% (*P* =.79), target vessel–related myocardial infarction occurred in 2.2%, 4.7%, and 3.7%, (*P* =.10), and clinically driven target lesion revascularization was required in 2.1%, 3.1%, and 0%, respectively (*P* =.15). Compared with those from high-recruiting sites, results from low-recruiting sites were similar for target lesion failure, major adverse cardiac events, stent thrombosis, and mortality, with a nonsignificant trend for higher rates of myocardial infarction.

**Conclusions:**

Despite regional differences in patient characteristics, the clinical outcomes between Supreme DES and durable polymer everolimus-eluting stent in the PIONEER III trial were not different, supporting the generalizability and robustness of the findings from this multicenter controlled trial.

## Highlights

• European patients had higher rates of non–ST-segment elevation myocardial infarction.
• Target lesion failure was consistent irrespective of geography.
• 1-Year target lesion failure was consistent in low- and high-recruiting sites.

## Central Illustration

**Figure:**
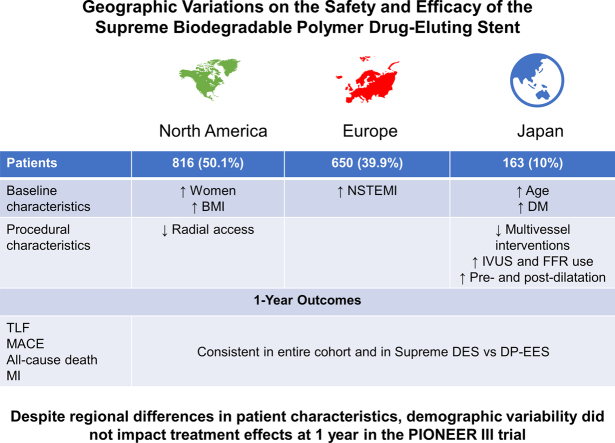

## Introduction

Percutaneous coronary intervention (PCI) with drug-eluting stents (DES) is the most frequently used method for coronary revascularization. Second-generation DES are now available that have more biocompatible polymer coatings, thinner struts, and a better safety profile than first-generation DES.^1^ Biodegradable polymer DES were developed to reduce polymer-related adverse effects known to be associated with delayed vessel healing and higher rates of stent thrombosis.^2^

The Supreme sirolimus-eluting stent (SES; SINOMED) consists of a thin-strut (80 μm) cobalt–chromium platform with a submicrometer thin electrografted base layer, interdigitated to a uniform biodegradable top-coat that releases sirolimus within 28 days.^3^ The PIONEER II OCT trial demonstrated that at 1 month, the Supreme DES had more complete strut coverage than the durable polymer everolimus-eluting stent (DP-EES) (XIENCE; Abbott Vascular),^4^ which elutes its antiproliferative drug over a longer timeframe. The pivotal PIONEER III IDE trial demonstrated that in patients with both acute coronary syndrome (ACS) and chronic coronary syndrome (CCS), the Supreme DES performance was noninferior to that of the standard DP-EES (target lesion failure [TLF] 5.4% with Supreme DES and 5.1% with DP-EES; absolute risk difference, 0.32% [95% CI, −1.87 to 2.5]; *P*_noninferiority_ =.002).^5^

Regional differences in patient demographics, operator expertise and technique, use of adjunctive pharmacotherapy, and other factors may affect clinical outcomes. We sought to evaluate regional differences in risk factors, procedural performance, and the overall and relative outcomes after Supreme DES or DP-EES implantation among patients recruited from North America, Europe, and Japan in the International PIONEER trial. Our aim was to determine whether outcomes remained consistent despite known geographic variations in patient characteristics and clinical practice and site recruitment volumes.

## Methods

### Study design

This is a subgroup analysis of the PIONEER III trial (NCT03168776) based on geographic regions, comparing North America, Europe, and Japan and the volume of recruitment at clinical sites. The PIONEER III trial design and rationale along with the principal 1-year results have been reported previously.^5^ In brief, PIONEER III was a prospective, international, 2:1 randomized, single-blind trial that compared the safety and effectiveness of PCI using the Supreme cobalt–chromium SES vs DP cobalt–chromium EES platform in patients with coronary artery disease presenting with ACS or CCS. Patients were recruited from 74 sites in the United States, Canada, Europe, and Japan. Inclusion criteria were presence of symptomatic ischemic heart disease with evidence of ischemia, unstable angina, or non–ST-segment elevation myocardial infarction (NSTEMI) requiring elective or urgent PCI, along with a de novo target vessel with a reference diameter of ≥2.25 mm and ≤4.0 mm and a visual diameter stenosis of ≥50% and <100%. Unprotected left main coronary artery disease, STEMI, and cardiogenic shock represented the main criteria for exclusion. Complete details of the inclusion and exclusion criteria have been reported.^5^ The trial was conducted according to the Declaration of Helsinki and Good Clinical Practice and was approved by the investigational review board or research ethics committee at each participating site. All participants gave their informed consent. Randomization was stratified according to recruiting site, clinical presentation (ACS vs CCS), and diabetes mellitus. Procedural recommendations were per standard of care. Daily aspirin was mandatory in all patients. An adenosine diphosphate platelet receptor antagonist was initiated prior to PCI and was continued for at least 6 months in patients with CCS and at least 12 months in patients with ACS in accordance with guidelines.^6^^,^^7^ Clinical follow-up was performed at 1 month, 6 months, 1 year, and is planned annually up to 5 years.

### End points

The primary end point was TLF, a composite of cardiac death, target vessel–related myocardial infarction (MI), or clinically driven target lesion revascularization (TLR) at 12 months. Major powered secondary end points included components of the primary end point. All patients were followed up to 1 year. The definitions of all end points have been reported previously.^5^ An independent clinical event committee (Cardiovascular Research Foundation, New York, NY) adjudicated all events after a review of original source documents. An independent angiographic core laboratory reviewed all angiograms (Yale Cardiovascular Research Center, New Haven, CT).

### Statistical analysis

Patient and procedural characteristics and outcomes were compared by region (North America, Europe, and Japan) and between high-volume sites (defined as sites recruiting ≥20 patients) and low-volume sites (recruiting <20 patients).

Discrete variables, expressed as percentages with frequencies, were compared using the χ^2^ or Fisher exact tests. Continuous variables, reported as mean ± standard deviation, were compared by *t* test if normally distributed or the Wilcoxon rank sum test if nonparametrically distributed. Event rates were based on Kaplan-Meier estimates in time-to-first-event analyses. Hazard ratios with 95% CIs were determined by the Cox regression analysis, and event rates were compared using the log-rank test. Interaction testing was performed to determine whether the relative risk of the major outcome measures at 1 year after Supreme DES vs DP-EES varied by geographic locations. All analyses were performed in the intention-to-treat population. A 2-sided *P* value of <.05 was considered statistically significant. All statistical analyses were performed with SAS software (version 9.4; SAS Institute).

## Results

Between October 2017 and July 2019, 1629 patients with coronary artery disease were randomized to the Supreme DES (n = 1086) and DP-EES (n = 543) groups. Of those, 816 (50.1%) patients were recruited at 37 North American sites (United States, n = 710; Canada, n = 106) and 650 (39.9%) were recruited at 29 European sites (Spain, n = 205; the Netherlands, n = 186; Belgium, n = 115; United Kingdom, n = 70; France, n = 55; and Switzerland, n = 19). The remaining 163 patients (10%) were recruited from 14 sites in Japan.

### Baseline patient characteristics and procedures by regions

The North American cohort comprised more women, patients with higher body mass index, and more patients with hypertension, previous coronary artery bypass grafting, multivessel and left anterior descending disease, and more unstable angina on presentation compared with those in the European and Japanese cohorts (Table 1). The baseline demographic and angiographic characteristics in patients randomized to the Supreme DES and DP-EES groups within each region were well balanced (Supplemental Tables S1-S3).

**Table 1:** Baseline demographic and angiographic characteristics according to the region of recruitment.

	North America (n = 816)	Europe (n = 650)	Japan (n = 163)	Overall (N = 1629)	*P*
Age, y	64.1 ± 9.7	63.4 ± 10.1	69.0 ± 9.5	64.3 ± 10.0	<.0001
Male sex	588/816 (72.1)	498/649 (76.7)	136/163 (83.4)	1222/1628 (75.1)	.004
Body mass index, kg/m^2^	30.7 ± 6.1	28.4 ± 4.6	24.3 ± 3.6	29.2 ± 5.7	<.0001
Hyperlipidemia	676/816 (82.8)	439/649 (67.6)	135/163 (82.8)	1250/1628 (76.8)	<.0001
Hypertension	659/816 (80.8)	399/649 (61.5)	127/163 (77.9)	1185/1628 (72.8)	<.0001
Diabetes	280/816 (34.3)	142/649 (21.9)	72/163 (44.2)	494/1628 (30.3)	<.0001
Current smoking	144/816 (17.6)	190/649 (29.3)	27/163 (16.6)	361/1628 (22.2)	<.0001
Previous myocardial infarction	145/816 (17.8)	109/649 (16.8)	36/163 (22.1)	290/1628 (17.8)	.29
Previous percutaneous coronary intervention	251/816 (30.8)	140/649 (21.6)	79/163 (48.5)	470/1628 (28.9)	<.0001
Target vessel percutaneous coronary intervention	46/251 (18.3)	26/140 (18.6)	17/79 (21.5)	89/470 (18.9)	.81
Previous coronary artery bypass grafting	54/816 (6.6)	20/649 (3.1)	2/163 (1.2)	76/1628 (4.7)	.0006
Previous stroke	28/816 (3.4)	23/649 (3.5)	14/163 (8.6)	65/1628 (4.0)	.007
History of congestive heart failure	42/816 (5.1)	15/649 (2.3)	17/163 (10.4)	74/1628 (4.5)	<.0001
Atrial fibrillation	22/816 (2.7)	10/649 (1.5)	3/163 (1.8)	35/1628 (2.1)	.30
Carotid disease	49/816 (6.0)	8/648 (1.2)	14/163 (8.6)	71/1627 (4.4)	<.0001
Peripheral artery disease	41/816 (5.0)	37/649 (5.7)	6/163 (3.7)	84/1628 (5.2)	.56
Ejection fraction, %	56.4 ± 8.7	58.4 ± 8.7	61.1 ± 10.0	57.6 ± 9.0	<.0001
Clinical presentation					
Stable angina	415/816 (50.9)	292/649 (45.0)	98/163 (60.1)	805/1628 (49.4)	.001
Unstable angina	210/816 (25.7)	98/649 (15.1)	24/163 (14.7)	332/1628 (20.4)	<.0001
Braunwald classification					
I	51/210 (24.3)	48/98 (49.0)	7/24 (29.2)	106/332 (31.9)	<.0001
II	54/210 (25.7)	29/98 (29.6)	14/24 (58.3)	97/332 (29.2)	.004
III	105/210 (50.0)	21/98 (21.4)	3/24 (12.5)	129/332 (38.9)	<.0001
Silent ischemia	37/816 (4.5)	75/649 (11.6)	38/163 (23.3)	150/1628 (9.2)	<.0001
Non–ST-segment elevation myocardial infarction	154/816 (18.9)	184/649 (28.4)	3/163 (1.8)	341/1628 (20.9)	<.0001
No. of diseased vessels^a^					
1	488/816 (59.8)	516/649 (79.5)	159/163 (97.5)	1163/1628 (71.4)	<.0001
2	220/816 (27.0)	117/649 (18.0)	4/163 (2.5)	341/1628 (20.9)	<.0001
3	92/816 (11.3)	15/649 (2.3)	0/163 (0.0)	107/1628 (6.6)	<.0001
≥4	16/816 (2.0)	1/649 (0.2)	0/163 (0.0)	17/1628 (1.0)	.001
Diseased vessels					
LAD/diagonal	513/816 (62.9)	349/649 (53.8)	89/163 (54.6)	951/1628 (58.4)	.001
LCX/OM/ramus	328/816 (40.2)	220/649 (33.9)	35/163 (21.5)	583/1628 (35.8)	<.0001
RCA/RPDA/RPL	403/816 (49.4)	228/649 (35.1)	43/163 (26.4)	674/1628 (41.4)	<.0001
Left main	16/816 (2.0)	1/649 (0.2)	0/163 (0.0)	17/1628 (1.0)	.001
Bypass vessel	10/816 (1.2)	2/649 (0.3)	0/163 (0.0)	12/1628 (0.7)	.06
Diameter stenosis, %	84.5 ± 9.4	83.6 ± 10.3	86.0 ± 8.8	84.3 ± 9.8	.007
Reference vessel diameter, mm	2.92 ± 0.49	2.95 ± 0.43	2.95 ± 0.43	2.93 ± 0.46	.30
P2Y12 loading dose administered					
Clopidogrel	366/818 (44.7)	221/649 (34.1)	4/163 (2.5)	591/1630 (36.3)	<.0001
Ticlopidine	5/818 (0.6)	2/649 (0.3)	0/163 (0.0)	7/1630 (0.4)	.46
Prasugrel	35/818 (4.3)	4/649 (0.6)	15/163 (9.2)	54/1630 (3.3)	<.0001
Ticagrelor	309/818 (37.8)	141/649 (21.7)	0/163 (0.0)	450/1630 (27.6)	<.0001

Values are n/N (%) or mean ± standard deviation.

LAD, left anterior descending; LCX, left circumflex; OM, obtuse marginal; RCA, right coronary artery; RPDA, right posterior descending artery; RPL, right posterolateral.

a Diameter stenosis of >50% by quantitative coronary angiography.

In Japan, patients underwent less multivessel interventions, fewer lesions were treated with fewer stents, and lesions were more likely to have predilation and postdilation performed compared with PCI procedures performed in Europe and North America (Table 2). In the North American cohort, there were fewer PCIs using a radial access but more intravascular ultrasound (IVUS) than fractional flow reserve (FFR) evaluations. Japan had the highest IVUS use compared with North America and Europe.

**Table 2:** Procedural characteristics according to the region of recruitment

	North America (n = 816)	Europe (n = 650)	Japan (n = 163)	Overall (N = 1629)	*P*
Vascular access site					
Radial	547/816 (67.0)	608/649 (93.7)	153/163 (93.9)	1308/1628 (80.3)	<.0001
Femoral	269/816 (33.0)	39/649 (6.0)	4/163 (2.5)	312/1628 (19.2)	<.0001
Brachial	0/816 (0.0)	2/649 (0.3)	6/163 (3.7)	8/1628 (0.5)	<.0001
No. of treated vessels					
1	720/816 (88.2)	555/649 (85.5)	157/163 (96.3)	1432/1628 (88.0)	.0007
2	95/816 (11.6)	93/649 (14.3)	6/163 (3.7)	194/1628 (11.9)	.0008
3	1/816 (0.1)	1/649 (0.2)	0/163 (0.0)	2/1628 (0.1)	.88
Target vessel(s)	n = 985	n = 814	n = 182	N = 1981	
LAD/diagonal	418/985 (42.4)	372/814 (45.7)	96/182 (52.7)	886/1981 (44.7)	.03
LCX/OM/ramus	255/985 (25.9)	212/814 (26.0)	37/182 (20.3)	504/1981 (25.4)	.25
RCA/RPDA/RPL	311/985 (31.6)	230/814 (28.3)	49/182 (26.9)	590/1981 (29.8)	.21
Left main	1/985 (0.1)	0/814 (0.0)	0/182 (0.0)	1/1981 (0.1)	.60
No. of target lesions	1.2 ± 0.5	1.3 ± 0.5	1.1 ± 0.3	1.2 ± 0.5	.001
Predilation of target vessel	730/985 (74.1)	599/814 (73.6)	178/182 (97.8)	1507/1981 (76.1)	<.0001
No. of stents implanted per target lesion	1.1 ± 0.3	1.1 ± 0.3	1.0 ± 0.3	1.1 ± 0.3	.02
Maximal stent diameter, mm	2.9 ± 0.4	3.0 ± 0.4	3.0 ± 0.4	2.9 ± 0.4	.09
Total stent length, mm	21.7 ± 8.9	22.4 ± 9.1	24.0 ± 7.7	22.2 ± 8.9	.006
Stent after dilation	506/981 (51.6)	375/810 (46.3)	150/178 (84.3)	1031/1969 (52.4)	<.0001
Use of fractional flow reserve	48/985 (4.9)	60/814 (7.4)	45/182 (24.7)	153/1981 (7.7)	<.0001
Use of intravascular ultrasound	146/985 (14.8)	17/814 (2.1)	137/182 (75.3)	300/1981 (15.1)	<.0001
Procedural duration, min	47 ± 27	45 ± 27	79 ± 304	50 ± 100	.0003
Procedural antiplatelet medications					
Glycoprotein IIb/IIIa inhibitors	76/818 (9.3)	8/649 (1.2)	0/163 (0.0)	84/1630 (5.2)	<.0001
Aspirin loading dose administered	587/818 (71.8)	164/649 (25.3)	15/163 (9.2)	766/1630 (47.0)	<.0001
Cangrelor	18/818 (2.2)	1/649 (0.2)	0/163 (0.0)	19/1630 (1.2)	.0005
Lesion success^a^	953/954 (99.9)	794/797 (99.6)	177/180 (98.3)	1924/1931 (99.6)	.03
Device success^b^	920/957 (96.1)	759/796 (95.4)	172/179 (96.1)	1851/1932 (95.8)	.87
Procedural success^c^	785/805 (97.5)	614/643 (95.5)	157/163 (96.3)	1556/1611 (96.6)	.17

Values are n/N (%) or mean ± standard deviation.

LAD, left anterior descending; LCX, left circumflex; OM, obtuse marginal; RCA, right coronary artery; RPDA, right posterior descending artery; RPL, right posterolateral.

a Attainment of final in-stent residual diameter stenosis of <30% by quantitative coronary angiography (QCA) using any percutaneous method.

b Attainment of final in-stent residual diameter stenosis of <30% of the target lesion (by QCA) using the assigned device. The analysis included all target lesions in the denominator, whether an attempt to implant the assigned device was made. Lesions treated with multiple stents in which 1 stent is an assigned device and >1 is not the assigned device are considered as device failures.

c Lesion success without the occurrence of in-hospital major adverse cardiac events, defined as a composite of all-cause death, myocardial infarction, or target vessel revascularization during the hospital stay.

Medication use varied by region. North American sites used more potent antiplatelet agents with more frequent P2Y12 loading before PCI and more frequent use of IIb/IIIa inhibitors compared with that in the other regions (Tables 1 and 2). Lesion, device, and procedural success rates were not different between the regions (Table 2).

### Baseline patient characteristics and procedures by site recruitment volume

Patients recruited from low-recruiting sites had higher rates of diabetes, hypertension, dyslipidemia, and previous PCI (Supplemental Table S4). Compared with those in high-recruiting sites, PCI procedures in low-recruiting sites comprised more predilation and postdilation, with more FFR and IVUS use (Supplemental Table S5). Lesion, device, and procedural success was not significantly different between low-recruiting and high-recruiting sites (Supplemental Table S5).

### Outcomes by region

In the entire cohort, there was a trend (nonsignificant) for higher rates of in-hospital and 1-month TLF among patients recruited from European sites (Table 3). This was predominantly driven by increased rates of periprocedural MI, most of which were NSTEMI. In patients treated using Supreme DES, the in-hospital and 1-month TLF and its individual components did not vary substantially with respect to the region of recruitment.

**Table 3:** Outcomes by region of recruitment

	North America		Europe		Japan		*P*	
	Supreme (n = 549)	All (n = 816)	Supreme (n = 429)	All (n = 650)	Supreme (n = 108)	All (n = 163)	Supreme	All
In-hospital								
Target lesion failure^a^	10 (1.8)	17 (2.1)	13 (3.0)	25 (3.8)	3 (2.8)	3 (1.8)	.45	.09
Major adverse cardiac events^b^	11 (2.0)	19 (2.3)	14 (3.3)	26 (4.0)	3 (2.8)	3 (1.8)	.46	.12
All-cause death	0 (0.0)	0 (0.0)	0 (0.0)	0 (0.0)	0 (0.0)	0 (0.0)	—	—
All MI	11 (2.0)	19 (2.3)	14 (3.3)	26 (4.0)	3 (2.8)	3 (1.8)	.46	.12
Target-vessel MI	10 (1.8)	17 (2.1)	13 (3.0)	25 (3.8)	3 (2.8)	3 (1.8)	.45	.09
Periprocedural MI	10 (1.8)	18 (2.2)	13 (3.0)	24 (3.7)	3 (2.8)	3 (1.8)	.45	.17
Clinically driven TLR	1 (0.2)	1 (0.1)	1 (0.2)	1 (0.2)	0 (0.0)	0 (0.0)	.88	.88
Acute stent thrombosis^c^	0 (0.0)	0 (0.0)	1 (0.2)	2 (0.3)	0 (0.0)	0 (0.0)	.46	.22
Definite	0 (0.0)	0 (0.0)	1 (0.2)	2 (0.3)	0 (0.0)	0 (0.0)	.46	.22
Probable	0 (0.0)	0 (0.0)	0 (0.0)	0 (0.0)	0 (0.0)	0 (0.0)	–	–
Possible	0 (0.0)	0 (0.0)	0 (0.0)	0 (0.0)	0 (0.0)	0 (0.0)	–	–
1 mo								
Target lesion failure^a^	14 (2.6)	22 (2.7)	17 (4.0)	30 (4.6)	3 (2.8)	3 (1.8)	.44	.07
Major adverse cardiac events^b^	16 (2.9)	25 (3.1)	20 (4.7)	33 (5.1)	3 (2.8)	3 (1.8)	.31	.052
All-cause death	1 (0.2)	2 (0.2)	2 (0.5)	2 (0.3)	0 (0.0)	0 (0.0)	.59	.78
Cardiovascular death	1 (0.2)	2 (0.2)	2 (0.5)	2 (0.3)	0 (0.0)	0 (0.0)	.59	.78
All MI	15 (2.7)	23 (2.8)	18 (4.2)	31 (4.8)	3 (2.8)	3 (1.8)	.42	.06
Target-vessel MI	11 (2.0)	18 (2.2)	16 (3.7)	29 (4.5)	3 (2.8)	3 (1.8)	.26	.029
Periprocedural MI	9 (1.6)	16 (2.0)	12 (2.8)	23 (3.5)	3 (2.8)	3 (1.8)	.43	.14
STEMI	3 (0.5)	3 (0.4)	3 (0.7)	5 (0.8)	0 (0.0)	0 (0.0)	.68	.35
NSTEMI	8 (1.5)	15 (1.8)	14 (3.3)	25 (3.8)	3 (2.8)	3 (1.8)	.16	.047
Clinically driven TLR	3 (0.5)	3 (0.4)	5 (1.2)	5 (0.8)	0 (0.0)	0 (0.0)	.34	.35
Early stent thrombosis^d^	2 (0.4)	3 (0.4)	5 (1.2)	6 (0.9)	0 (0.0)	0 (0.0)	.20	.22
Definite	1 (0.2)	2 (0.2)	4 (0.9)	5 (0.8)	0 (0.0)	0 (0.0)	.17	.21
Probable	1 (0.2)	1 (0.1)	1 (0.2)	1 (0.2)	0 (0.0)	0 (0.0)	.88	.88
Possible	0 (0.0)	0 (0.0)	0 (0.0)	0 (0.0)	0 (0.0)	0 (0.0)	–	–
6 mo								
Target lesion failure^a^	18 (3.3)	30 (3.7)	19 (4.4)	32 (4.9)	4 (3.7)	4 (2.5)	.64	.26
Major adverse cardiac events^b^	31 (5.7)	49 (6.0)	23 (5.4)	37 (5.7)	4 (3.7)	3.1 (5)	.72	.33
All-cause death	3 (0.5)	7 (0.9)	2 (0.5)	2 (0.3)	0 (0.0)	1 (0.6)	.74	.41
Cardiovascular death	2 (0.4)	5 (0.6)	2 (0.5)	2 (0.3)	0 (0.0)	0 (0.0)	.77	.46
All MI	18 (3.3)	27 (3.3)	21 (4.9)	34 (5.2)	4 (3.7)	4 (2.5)	.43	.10
Target-vessel MI	11 (2.0)	19 (2.3)	18 (4.2)	31 (4.8)	4 (3.7)	4 (2.5)	.13	.03
STEMI	3 (0.5)	3 (0.4)	4 (0.9)	6 (0.9)	0 (0.0)	0 (0.0)	.51	.22
NSTEMI	8 (1.5)	16 (2.0)	15 (3.5)	26 (4.0)	4 (3.7)	4 (2.5)	.09	.06
Clinically driven TLR	6 (1.1)	7 (0.9)	7 (1.6)	8 (1.2)	0 (0.0)	0 (0.0)	.36	.33
1 y								
Target lesion failure^a^	24 (4.4)	38 (4.7)	29 (6.8)	42 (6.5)	4 (3.7)	4 (2.5)	.20	.08
Major adverse cardiac events^b^	41 (7.6)	63 (7.8)	34 (8.0)	50 (7.7)	4 (3.7)	5 (3.1)	.31	.10
All-cause death	4 (0.7)	10 (1.3)	2 (0.5)	3 (0.5)	0 (0.0)	1 (0.6)	.62	.27
Cardiovascular death	2 (0.4)	6 (0.8)	2 (0.5)	2 (0.3)	0 (0.0)	0 (0.0)	.77	.32
All MI	21 (3.9)	32 (4.0)	26 (6.1)	39 (6.0)	4 (3.7)	4 (2.5)	.23	.07
Target-vessel MI	12 (2.2)	21 (2.6)	20 (4.7)	33 (5.1)	4 (3.7)	4 (2.5)	.10	.03
STEMI	3 (0.5)	5 (0.6)	5 (1.2)	7 (1.1)	0 (0.0)	0 (0.0)	.34	.30
NSTEMI	9 (1.7)	17 (2.1)	16 (3.7)	27 (4.2)	4 (3.7)	4 (2.5)	.11	.06
Clinically driven TLR	11 (2.0)	15 (1.8)	13 (3.1)	14 (2.2)	0 (0.0)	0 (0.0)	.15	.18
Late stent thrombosis^e^	0 (0.0)	2 (0.3)	1 (0.2)	1 (0.2)	0 (0.0)	0 (0.0)	.47	.78
Definite	0 (0.0)	0 (0.0)	1 (0.2)	1 (0.2)	0 (0.0)	0 (0.0)	.47	.48
Probable	0 (0.0)	0 (0.0)	0 (0.0)	0 (0.0)	0 (0.0)	0 (0.0)	–	–
Possible	0 (0.0)	2 (0.3)	0 (0.0)	0 (0.0)	0 (0.0)	0 (0.0)	–	.37

Values are n (%).

MI, myocardial infarction; NSTEMI, non–ST-segment elevation MI; STEMI, ST-segment elevation MI; TLR, target lesion revascularization.

a Composite of cardiac death, target vessel–related MI, or clinically driven TLR.

b Composite of all-cause death, MI, or target vessel revascularization.

c ≤1 day after stent implantation.

d 0 to 30 days after stent implantation.

e >30 days to 1 year after stent implantation.

**Figure 1:**
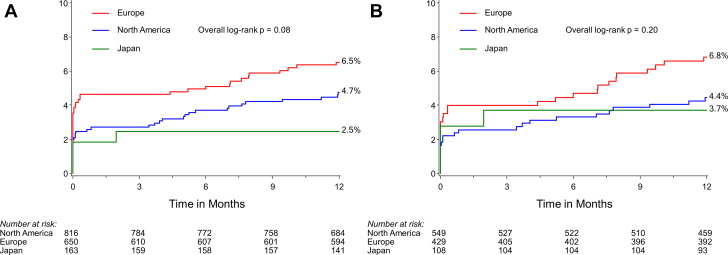
**Rates of target lesion failure at 12 months by the region of recruitment.** (**A**) Target lesion failure by site of recruitment (y-axis). (**B**) Target lesion failure by the site of recruitment in the Supreme DES cohort (y-axis). Target lesion failure is a composite of cardiac death, target vessel–related myocardial infarction, or clinically driven target lesion revascularization. DES, drug-eluting stents.

**Figure 2:**
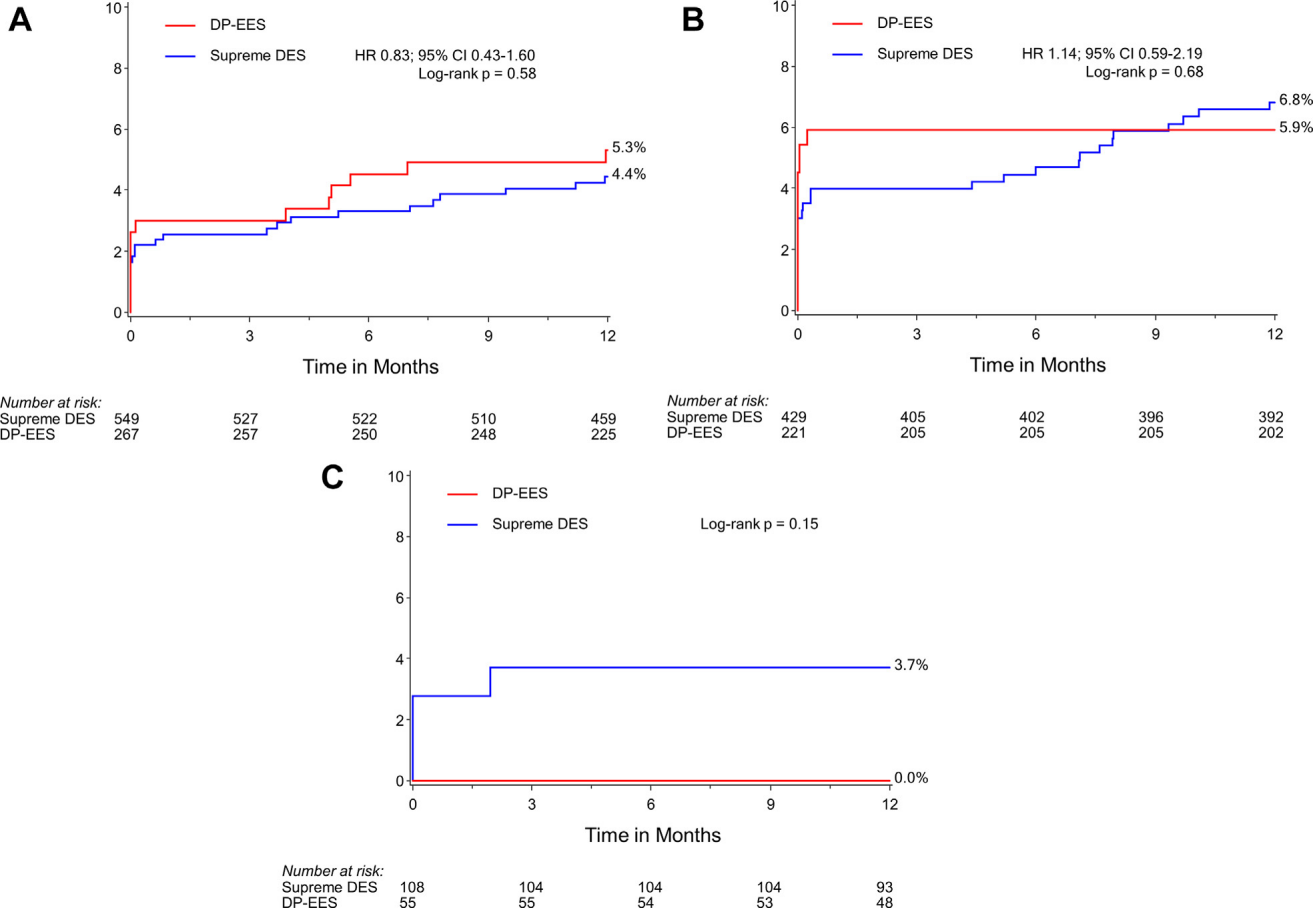
**Rates of target lesion failure at 12 months by region of recruitment and stent used.** Target lesion failure in Supreme DES vs DP-EES (y-axis) at sites in (**A**) North America, (**B**) Europe, and (**C**) Japan. Target lesion failure is a composite of cardiac death, target vessel–related myocardial infarction, or clinically driven target lesion revascularization. DES, drug-eluting stents; DP-EES, durable polymer everolimus-eluting stents; HR, hazard ratio.

At 1 year, mortality rates were similar by region, but MI was more common among patients recruited in Europe than among patients recruited in North America and Japan, resulting in a trend for TLF (*P* =.08) and major adverse cardiac events (MACE; composite of all-cause death, MI, or target vessel revascularization; *P* =.10) (Table 3 and Figure 1). Among patients treated with Supreme DES, 1-year TLF and its components were similar in the North American, European, and Japanese cohorts. The relative risks for 1-year TLF after Supreme DES vs DP-EES implantation were consistent irrespective of geography (Figure 2). There was no interaction between groups for major outcome measures by geographic location (Central Illustration).

**Central Illustration:**
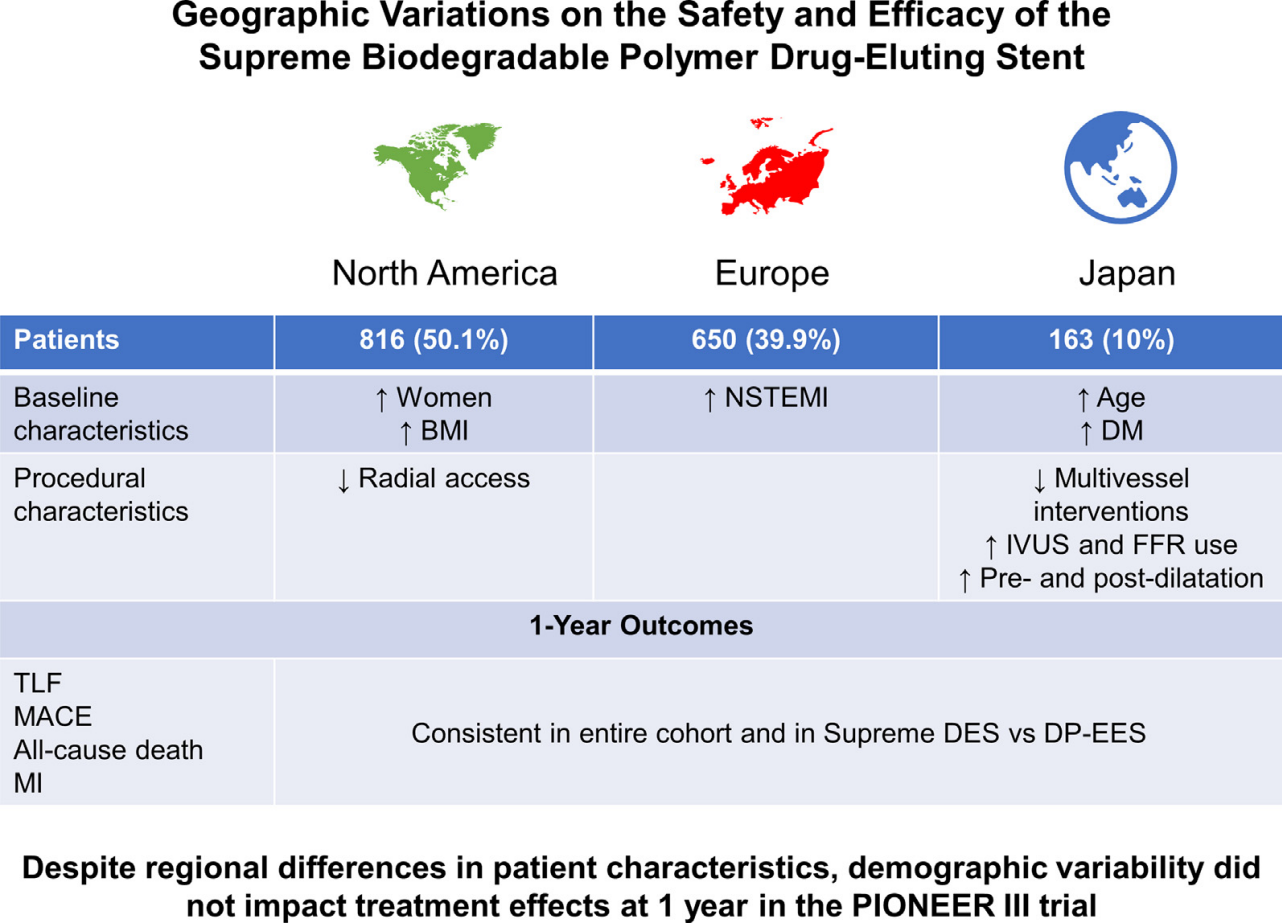
**Geographic variations on the safety and efficacy of Supreme biodegradable polymer drug-eluting stents.** BMI, body mass index; DES, drug-eluting stent; DM, diabetes mellitus; DP-EES, durable polymer everolimus-eluting stents; FFR, fractional flow reserve; IVUS, intravascular ultrasound; MACE, major adverse cardiac events; MI, myocardial infarction; NSTEMI, non–ST-segment elevation MI; TLF, target lesion failure.

**Figure 3:**
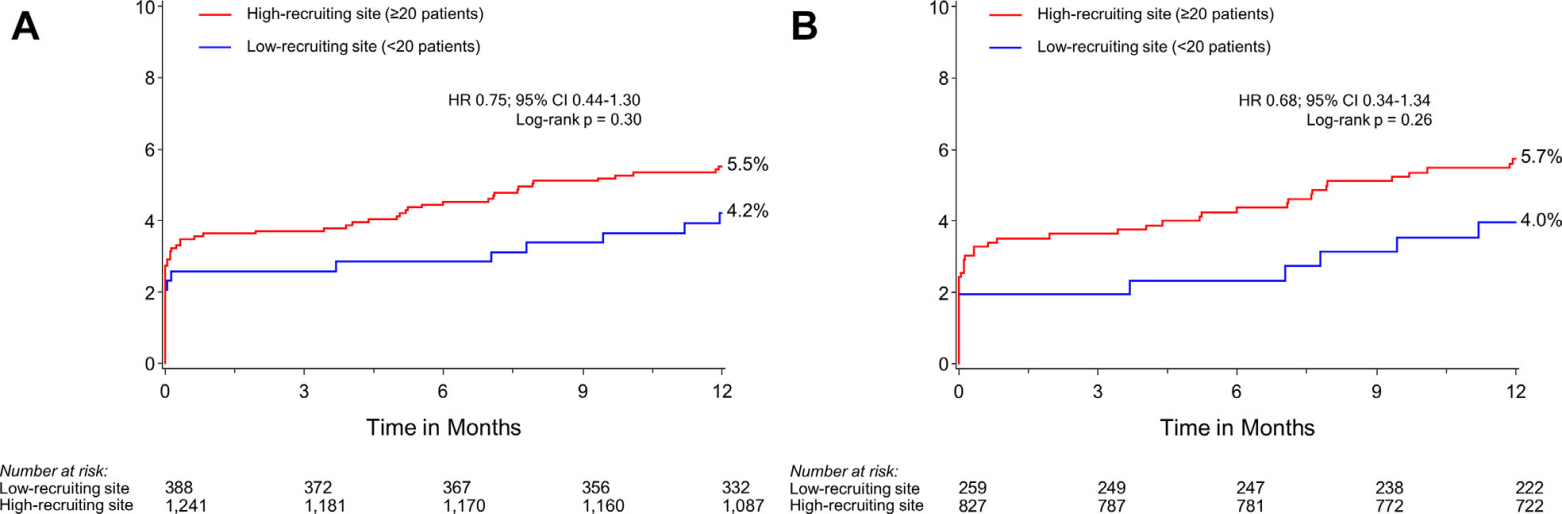
**Rates of target lesion failure at 12 months in high- vs low-recruiting sites.** (**A**) Target lesion failure at high- vs low-recruiting sites (y-axis). (**B**) Target lesion failure at high- vs low-recruiting sites in the Supreme DES cohort (y-axis). High-recruiting sites recruited ≥20 patients. Low-recruiting sites recruited <20 patients. Target lesion failure is a composite of cardiac death, target vessel–related myocardial infarction, or clinically driven target lesion revascularization. DES, drug-eluting stents; HR, hazard ratio.

Low- and high-recruiting sites had similar rates of TLF, MACE, and mortality, but low-recruiting sites had a trend for higher rates of MI; stent thrombosis rates were similar among regions (Table 4 and Figure 3).

**Table 4:** Outcomes at 1 year by recruitment volume

	Low-recruiting sites (n = 388)	High-recruiting sites (n = 1241)	All sites (N = 1629)	Hazard ratio (95% CI)^a^	*P*^b^
Target lesion failure^c^	16 (4.2)	68 (5.5)	84 (5.2)	0.75 (0.44-1.30)	.30
Major adverse cardiac events^d^	22 (5.8)	96 (7.8)	118 (7.3)	0.73 (0.46-1.16)	.18
All-cause death	4 (1.0)	10 (0.8)	14 (0.9)	1.30 (0.41-4.13)	.66
Cardiovascular death	1 (0.3)	7 (0.6)	8 (0.5)	0.46 (0.06-3.74)	.46
All MI	11 (2.9)	64 (5.2)	75 (4.6)	0.55 (0.29-1.04)	.06
Target-vessel MI	9 (2.3)	49 (4.0)	58 (3.6)	0.59 (0.29-1.20)	.13
STEMI	0 (0.0)	12 (1.0)	12 (0.7)	–	.053
NSTEMI	11 (2.9)	53 (4.3)	64 (4.0)	0.66 (0.35-1.27)	.21
Clinically driven TLR	6 (1.6)	23 (1.9)	29 (1.8)	0.84 (0.34-2.07)	.71
Stent thrombosis	2 (0.5)	10 (0.8)	12 (0.7)	0.64 (0.14-2.93)	.56
Definite	2 (0.5)	6 (0.5)	8 (0.5)	1.07 (0.22-5.30)	.94
Probable	0 (0.0)	2 (0.2)	2 (0.1)	–	.43
Possible	0 (0.0)	2 (0.2)	2 (0.1)	–	.43

Values are n (%).

MI, myocardial infarction; NSTEMI, non–ST-segment elevation MI; STEMI, ST-segment elevation MI; TLR, target lesion revascularization.

a Hazard ratio and 95% Wald CI are from Cox proportional hazards model, including treatment as a covariate.

b *P* values for treatment comparison are from the log-rank test.

c Composite of cardiac death, target vessel–related MI, or clinically driven target lesion revascularization.

d Composite of all-cause death, MI, or target vessel revascularization.

## Discussion

The main findings of this analysis are as follows. (1) The relative risk of the 1-year device-oriented composite TLF end point was consistent irrespective of geography both in the entire cohort and when comparing the Supreme DES with DP-EES cohort. (2) Individuals recruited from Europe had fewer comorbidities but more frequently presented with NSTEMI compared with patients recruited from North America and Japan. In addition, patients in Europe had higher rates of MI at 30 days (typically target vessel–related NSTEMI), which persisted at 1-year follow-up. (3) The relative risk for 1-year TLF was consistent in low- and high-recruiting sites. (4) Patients recruited in Japan had very few events at the 1-year follow-up (only 1 noncardiovascular death and 4 target vessel–related NSTEMI of the 163 patients).

This analysis of the PIONEER III trial illustrates the substantial variability in baseline characteristics, procedural techniques, and clinical outcomes among patients undergoing PCI in North America, Europe, and Japan. Patient risk factors, techniques, and outcomes of PCI are known to vary within and between North America, Europe, and Japan.^8^, ^9^, ^10^, ^11^ A recent review of 701 PCI trials between 1990 and 2014 indicated that North American trials recruited the lowest proportion of male participants but the highest proportion of individuals with hypertension and dyslipidemia, whereas Asia recruited the highest proportion of patients with diabetes.^11^ The population breakdown of PIONEER III was consistent with these previous observations. Because differences in patient characteristics by geography may also affect outcomes, Liu et al^11^ indicated that clinical trial results may not be applicable to patient populations from different regions. The present analysis demonstrated that the findings of the PIONEER III trial and the excellent safety and efficacy of the Supreme DES are relevant in North America, Europe, and Japan despite substantial different patient characteristics and procedural approaches.

At 1 year, the primary end point of TLF occurred in similar proportions of patients regardless of region. Importantly, patients from Europe experienced higher rates of NSTEMI at 30-day, 6-month, and 1-year follow-ups without differences in mortality, clinically driven TLR, or stent thrombosis. These findings are likely to be a function of the higher prevalence of NSTEMI on presentation and the more frequent multilesion and multivessel PCIs among the European cohort.^12^ The sensitivity of cardiac enzyme testing methods was consistent irrespective of geography. Furthermore, lower use of potent antiplatelet therapy (both preprocedural P2Y12 loading dose and per-procedure glycoprotein IIb/IIIa inhibitors), IVUS, and postdilation could be important factors associated with the higher incidence of target vessel–related MI in the European cohort.^13^^,^^14^ These factors cannot wholly account for the disparity in NSTEMI rates because the rates of lesion/device/procedure success did not differ significantly in North America, Europe, and Japan. Importantly, this higher rate of NSTEMI among Europeans was observed in the entire cohort of the PIONEER III trial but not in patients treated with the Supreme DES, indicating that this was not related to the investigated device but more likely to be associated with different patient presentation or procedure details.

Findings related to low- versus high-recruiting sites provide interesting additional information. Despite having fewer comorbidities and a higher prevalence of acute presentation compared with those from sites with lower volume recruitment, individuals recruited in high-recruiting sites had similar rates of TLF and MACE but a trend toward higher rates of MI at 1 year; these events were mainly STEMI and were not associated with higher rates of stent thrombosis. Notably, the extremely low event rates reported among Japanese patients is remarkable with only 4 target-vessel NSTEMI and 1 noncardiac death among 163 patients. No stent thrombosis and no TLR were reported from the Japanese cohort. The reasons for this are probably multifactorial. This cannot be attributed to favorable patient risk profile because Japanese sites recruited older patients with higher rates of comorbidities including diabetes mellitus (44.2%), previous PCI (48.5%), a history of congestive heart failure (10.4%), and a history of carotid disease and stroke (8.6% for both) at the time of randomization. However, Japanese patients had less acute presentations (83.4% of them had stable angina or silent ischemia), and most of them had single-vessel disease (97.5%), indicating a highly selected “ideal” population for the purpose of a randomized comparison. Moreover, procedural characteristics were significantly different from North American and European sites with the highest use of predilation and postdilation (97.8% and 84.3%, respectively) and PCI guidance with FFR (25%) and IVUS (75%) evaluation, which is typically protocolized in Japan. Furthermore, racial and genetic differences in bioavailability of adjunctive pharmacology and increased sensitivity to antiplatelet agents among Japanese patients may contribute to observed differences.

### Limitations

Recruiting site region was not a stratification variable in the PIONEER III trial. Although the baseline characteristics of patients randomized to the Supreme DES versus DP-EES cohorts within each geographic region were well balanced, differences in unmeasured confounders cannot be excluded. The regions of recruitment were subgroups and, hence, were individually underpowered for hypothesis testing. We relied on tests for interaction to examine whether relative outcomes for Supreme DES versus DP-EES use varied across the geographies; however, interaction testing was also underpowered. Therefore, we cannot exclude small differences in outcomes between regions. This is particularly true for the components of the primary end point. Of note, however, early MI (typically, periprocedural NSTEMI, ie, cardiac enzymes elevation) tended to be greater in European sites compared with that at North American and Japanese sites. It is unknown whether the trend for greater rate of clinically driven TLR contributed to this difference or was a consequence of it. The use of stress testing during follow-up and the exact indications for reintervention depended on the operators’ routine practice and geographical differences in clinical criteria that might account for regional differences in revascularization were not collected in detail in the PIONEER III trial. Further study is warranted to understand the greater rates of early NSTEMI in Europe, which were sustained at the 1-year follow-up. This cannot be entirely explained by European sites recruiting individuals with more complex coronary anatomy because patients recruited at North American and Japanese sites had greater rates of comorbidities (especially diabetes mellitus), previous PCI, and unstable angina on presentation. Additional studies are required to determine whether the numerous patient and procedural differences between Europe and North America translate into differences in early MI after complex or multivessel interventions. Only 163 patients were recruited from Japan (approximately 10% of the patients recruited in the trial), precluding meaningful comparisons of outcomes from this region. Moreover, the results do not apply to patients with complex PCI (total occlusions, unprotected left main lesion, a lesion located in an arterial or vein graft, in-stent restenosis, and 2-stent bifurcation lesions), who were excluded from the PIONEER III trial. Finally, this analysis reports the 1-year outcomes after Supreme DES versus DP-EES implantation, which precludes identifying any potential interaction between geography and Supreme DES technology in terms of late healing. An additional geographic analysis is warranted at the 5-year follow-up.

The high-/low-volume analysis has substantial limitations. First, the cutoff value of 20 patients recruited in the trial is arbitrary, and a different cutoff may yield different results. Second, a low recruitment volume in the trial does not necessarily imply a low-volume PCI center. Third, the recruitment volume in the trial may be different from a learning curve because multiple operators may have treated few patients in a single high-recruitment site, whereas a single operator may have treated more patients in a low-recruitment site.

## Conclusions

Geographic variability did not affect treatment effects at 1 year in the PIONEER III trial, supporting the generalizability and robustness of the findings from this multicenter randomized controlled trial.
