# Supplementary material for: Geographic Variations on the Safety and Efficacy of the Supreme Biodegradable Polymer DES: Results From PIONEER III
Source: J Soc Cardiovasc Angiogr Interv. 2022 Nov 25;2(1):100515. doi: 10.1016/j.jscai.2022.100515 (PMC11308104; doi:10.1016/j.jscai.2022.100515)
Supplement: Supplementary Data [file mmc1.docx]

**Supplemental Table 1. Baseline Demographic and Angiographic Characteristics of Patients Recruited in North America According to Randomization Arm**

|  | **Supreme**  **n = 549** | **DP-EES**  **n = 267** | **Overall**  **N= 816** | **P-Value** |
| --- | --- | --- | --- | --- |
| Age, years | 64.2±9.7 | 63.9±9.8 | 64.1±9.7 | 0.73 |
| Male sex | 404/549 (73.6%) | 184/267 (68.9%) | 588/816 (72.1%) | 0.16 |
| Body mass index, kg/m^2^ | 30.7 ±6.1 | 30.7 ±6.1 | 30.7 ±6.1 | 0.83 |
| Hyperlipidemia | 457/549 (83.2%) | 219/267 (82.0%) | 676/816 (82.8%) | 0.66 |
| Hypertension | 454/549 (82.7%) | 205/267 (76.8%) | 659/816 (80.8%) | 0.044 |
| Diabetes | 190/549 (34.6%) | 90/267 (33.7%) | 280/816 (34.3%) | 0.80 |
| Current smoking | 94/549 (17.1%) | 50/267 (18.7%) | 144/816 (17.6%) | 0.57 |
| Prior myocardial infarction | 99/549 (18.0%) | 46/267(17.2%) | 145/816 (17.8%) | 0.78 |
| Prior percutaneous coronary intervention | 172/549 (31.3%) | 79/267 (29.6%) | 251/816 (30.8%) | 0.61 |
| Target vessel percutaneous coronary intervention | 32/172 (18.6%) | 14/79 (17.7%) | 46/251 (18.3%) | 0.87 |
| Prior coronary artery bypass grafting | 37/549 (6.7%) | 17/267 (6.4%) | 54/816 (6.6%) | 0.84 |
| Prior stroke | 19/549 (3.5%) | 9/267 (3.4%) | 28/816 (3.4%) | 0.95 |
| History of congestive heart failure | 28/549 (5.1%) | 14/267 (5.2%) | 42/816 (5.1%) | 0.93 |
| Atrial fibrillation | 14/549 (2.6%) | 8/267 (3.0%) | 22/816 (2.7%) | 0.71 |
| Carotid disease | 32/549 (5.8%) | 17/267 (6.4%) | 49/816 (6.0%) | 0.76 |
| Peripheral artery disease | 26/549 (4.7%) | 15/267 (5.6%) | 41/816 (5.0%) | 0.59 |
| Ejection fraction, % | 56.3±8.9 | 56.5±8.2 | 56.4±8.7 | 0.95 |
| Clinical presentation |  |  |  |  |
| Stable angina | 276/549 (50.3%) | 139/267 (52.1%) | 415/816 (50.9%) | 0.63 |
| Unstable angina | 141/549 (25.7%) | 69/267 (25.8%) | 210/816 (25.7%) | 0.96 |
| Braunwald classification |  |  |  |  |
| I | 35/141 (24.8%) | 16/69 (23.2%) | 51/210 (24.3%) | 0.80 |
| II | 35/141 (24.8%) | 19/69 (27.5%) | 54/210 (25.7%) | 0.67 |
| III | 71/141 (50.4%) | 34/69 (49.3%) | 105/210 (50.0%) | 0.88 |
| Silent ischemia | 29/549 (5.3%) | 8/267 (3.0%) | 37/816 (4.5%) | 0.14 |
| NSTEMI | 103/549 (18.8%) | 51/267 (19.1%) | 154/816 (18.9%) | 0.91 |
| Number of diseased vessels* |  |  |  |  |
| 1 | 337/549 (61.4%) | 151/267 (56.6%) | 488/816 (59.8%) | 0.19 |
| 2 | 140/549 (25.5%) | 80/267 (30.0%) | 220/816 (27.0%) | 0.18 |
| 3 | 62/549 (11.3%) | 30/267 (11.2%) | 92/816 (11.3%) | 0.98 |
| ≥4 | 10/549 (1.8%) | 6/267 (2.2%) | 16/816 (2.0%) | 0.68 |
| Diseased vessels |  |  |  |  |
| LAD/diagonal | 345/549 (62.8%) | 168/267 (62.9%) | 513/816 (62.9%) | 0.98 |
| LCX/OM/ramus | 214/549 (39.0%) | 114/267 (42.7%) | 328/816 (40.2%) | 0.31 |
| RCA/RPDA/RPL | 267/549 (48.6%) | 136/267 (50.9%) | 403/816 (49.4%) | 0.54 |
| Left Main | 13/549 (2.4%) | 3/267 (1.1%) | 16/816 (2.0%) | 0.23 |
| Bypass vessel | 6/549 (1.1%) | 4/267 (1.5%) | 10/816 (1.2%) | 0.74 |
| Diameter stenosis, % | 84.5±9.4 | 84.6±9.4 | 84.5±9.4 | 0.83 |
| Reference vessel diameter, mm | 2.91±0.43 | 2.93±0.59 | 2.92±0.49 | 0.84 |

Data are n/N (%) or mean±standard deviation. *Diameter stenosis >50% by quantitative coronary angiography. NSTEMI = non-ST elevation myocardial infarction; LAD = left anterior descending; LCX = left circumflex; OM = obtuse marginal; RCA = right coronary artery; RPDA = right posterior descending artery; RPL = right postero-lateral.

**Supplemental Table 2. Baseline Demographic and Angiographic Characteristics of Patients Recruited in Europe According to Randomization Arm**

|  | **Supreme**  **n = 429** | **DP-EES**  **n = 221** | **Overall**  **N=650** | **P-Value** |
| --- | --- | --- | --- | --- |
| Age, years | 63.7±9.7 | 62.9±10.8 | 63.5±10.1 | 0.49 |
| Male sex | 337/429 (78.6%) | 161/220 (73.2%) | 498/649 (76.7%) | 0.13 |
| Body mass index, kg/m^2^ | 28.3±4.5 | 28.7±5.0 | 28.4±4.6 | 0.39 |
| Hyperlipidemia | 289/429 (67.4%) | 150/220 (68.2%) | 439/649 (67.6%) | 0.83 |
| Hypertension | 268/429 (62.5%) | 131/220 (59.5%) | 399/649 (61.5%) | 0.47 |
| Diabetes | 91/429 (21.2%) | 51/220 (23.2%) | 142/649 (21.9%) | 0.57 |
| Current smoking | 127/429 (29.6%) | 63/220 (28.6%) | 190/649 (29.3%) | 0.80 |
| Prior myocardial infarction | 72/429 (16.8%) | 37/220 (16.8%) | 109/649 (16.8%) | 0.99 |
| Prior percutaneous coronary intervention | 84/429 (19.6%) | 56/220 (25.5%) | 140/649 (21.6%) | 0.09 |
| Target vessel percutaneous coronary intervention | 14/84 (16.7%) | 12/56 (21.4%) | 26/140 (18.6%) | 0.48 |
| Prior coronary artery bypass grafting | 14/429 (3.3%) | 6/220 (2.7%) | 20/649 (3.1%) | 0.71 |
| Prior stroke | 15/429 (3.5%) | 8/220 (3.6%) | 23/649 (3.5%) | 0.93 |
| History of congestive heart failure | 10/429 (2.3%) | 5/220 (2.3%) | 15/649 (2.3%) | 0.96 |
| Atrial fibrillation | 5/429 (1.2%) | 5/220 (2.3%) | 10/649 (1.5%) | 0.32 |
| Carotid disease | 7/429 (1.6%) | 1/219 (0.5%) | 8/648 (1.2%) | 0.28 |
| Peripheral artery disease | 25/429 (5.8%) | 12/220 (5.5%) | 37/649 (5.7%) | 0.85 |
| Ejection fraction, % | 58.3±8.7 | 58.6±8.9 | 58.4±8.7 | 0.56 |
| Clinical presentation |  |  |  |  |
| Stable angina | 197/429 (45.9%) | 95/220 (43.2%) | 292/649 (45.0%) | 0.51 |
| Unstable angina | 62/429 (14.5%) | 36/220 (16.4%) | 98/649 (15.1%) | 0.52 |
| Braunwald classification |  |  |  |  |
| I | 27/62 (43.5%) | 21/36 (58.3%) | 48/98 (49.0%) | 0.16 |
| II | 18/62 (29.0%) | 11/36 (30.6%) | 29/98 (29.6%) | 0.87 |
| III | 17/62 (27.4%) | 4/36 (11.1%) | 21/98 (21.4%) | 0.058 |
| Silent ischemia | 53/429 (12.4%) | 22/220 (10.0%) | 75/649 (11.6%) | 0.37 |
| NSTEMI | 117/429 (27.3%) | 67/220 (30.5%) | 184/649 (28.4%) | 0.39 |
| Number of diseased vessels* |  |  |  |  |
| 1 | 346/429 (80.7%) | 170/220 (77.3%) | 516/649 (79.5%) | 0.31 |
| 2 | 70/429 (16.3%) | 47/220 (21.4%) | 117/649 (18.0%) | 0.11 |
| 3 | 13/429 (3.0%) | 2/220 (0.9%) | 15/649 (2.3%) | 0.09 |
| ≥4 | 0/429 (0.0%) | 1/220 (0.5%) | 1/649 (0.2%) | 0.34 |
| Diseased vessels |  |  |  |  |
| LAD/diagonal | 225/429 (52.4%) | 124/220 (56.4%) | 349/649 (53.8%) | 0.34 |
| LCX/OM/ramus | 155/429 (36.1%) | 65/220 (29.5%) | 220/649 (33.9%) | 0.09 |
| RCA/RPDA/RPL | 144/429 (33.6%) | 84/220 (38.2%) | 228/649 (35.1%) | 0.24 |
| Left Main | 0/429 (0.0%) | 1/220 (0.5%) | 1/649 (0.2%) | 0.34 |
| Bypass vessel | 1/429 (0.2%) | 1/220 (0.5%) | 2/649 (0.3%) | 1.0 |
| Diameter stenosis, % | 83.5±10.6 | 83.9±9.8 | 83.6±10.3 | 0.72 |
| Reference vessel diameter, mm | 2.95±0.43 | 2.95±0.44 | 2.95±0.43 | 0.89 |

Data are n/N (%) or mean±standard deviation. *Diameter stenosis >50% by quantitative coronary angiography. NSTEMI = non-ST elevation myocardial infarction; LAD = left anterior descending; LCX = left circumflex; OM = obtuse marginal; RCA = right coronary artery; RPDA = right posterior descending artery; RPL = right postero-lateral.

**Supplemental Table 3. Baseline Demographic and Angiographic Characteristics of Patients Recruited in Japan According to Randomization Arm**

|  | **Supreme**  **n = 108** | **DP-EES**  **n = 55** | **Overall**  **N= 163** | **P-Value** |
| --- | --- | --- | --- | --- |
| Age, years | 69.4±9.7 | 68.1±9.2 | 69.0±9.5 | 0.41 |
| Male sex | 87/108 (80.6%) | 49/55 (89.1%) | 136/163 (83.4%) | 0.17 |
| Body mass index, kg/m^2^ | 23.8±3.6 | 25.3±3.6 | 24.3±3.6 | 0.015 |
| Hyperlipidemia | 91/108 (84.3%) | 44/55 (80.0%) | 135/163 (82.8%) | 0.50 |
| Hypertension | 84/108 (77.8%) | 43/55 (78.2%) | 127/163 (77.9%) | 0.95 |
| Diabetes | 50/108 (46.3%) | 22/55 (40.0%) | 72/163 (44.2%) | 0.44 |
| Current smoking | 19/108 (17.6%) | 8/55 (14.5%) | 27/163 (16.6%) | 0.62 |
| Prior myocardial infarction | 18/108 (16.7%) | 18/55 (32.7%) | 36/163 (22.1%) | 0.019 |
| Prior percutaneous coronary intervention | 48/108 (44.4%) | 31/55 (56.4%) | 79/163 (48.5%) | 0.15 |
| Target vessel percutaneous coronary intervention | 12/48 (25.0%) | 5/31 (16.1%) | 17/79 (21.5%) | 0.35 |
| Prior coronary artery bypass grafting | 2/108 (1.9%) | 0/55 (0.0%) | 2/163 (1.2%) | 0.55 |
| Prior stroke | 12/108 (11.1%) | 2/55 (3.6%) | 14/163 (8.6%) | 0.14 |
| History of congestive heart failure | 13/108 (12.0%) | 4/55 (7.3%) | 17/163 (10.4%) | 0.35 |
| Atrial fibrillation | 3/108 (2.8%) | 0/55 (0.0%) | 3/163 (1.8%) | 0.55 |
| Carotid disease | 10/108 (9.3%) | 4/55 (7.3%) | 14/163 (8.6%) | 0.77 |
| Peripheral artery disease | 5/108 (4.6%) | 1/55 (1.8%) | 6/163 (3.7%) | 0.66 |
| Ejection fraction, % | 61.4±10.2 | 60.6±9.6 | 61.1±10.0 | 0.65 |
| Clinical presentation |  |  |  |  |
| Stable angina | 63/108 (58.3%) | 35/55 (63.6%) | 98/163 (60.1%) | 0.51 |
| Unstable angina | 15/108 (13.9%) | 9/55 (16.4%) | 24/163 (14.7%) | 0.67 |
| Braunwald classification |  |  |  |  |
| I | 4/15 (26.7%) | 3/9 (33.3%) | 7/24 (29.2%) | 1.0 |
| II | 8/15 (53.3%) | 6/9 (66.7%) | 14/24 (58.3%) | 0.68 |
| III | 3/15 (20.0%) | 0/9 (0.0%) | 3/24 (12.5%) | 0.27 |
| Silent ischemia | 27/108 (25.0%) | 11/55 (20.0%) | 38/163 (23.3%) | 0.48 |
| NSTEMI | 3/108 (2.8%) | 0/55 (0.0%) | 3/163 (1.8%) | 0.55 |
| Number of diseased vessels* |  |  |  |  |
| 1 | 105/108 (97.2%) | 54/55 (98.2%) | 159/163 (97.5%) | 1.0 |
| 2 | 3/108 (2.8%) | 1/55 (1.8%) | 4/163 (2.5%) | 1.0 |
| 3 | 0/108 (0.0%) | 0/55 (0.0%) | 0/163 (0.0%) | NA |
| ≥4 | 0/108 (0.0%) | 0/55 (0.0%) | 0/163 (0.0%) | NA |
| Diseased vessels |  |  |  |  |
| LAD/diagonal | 61/108 (56.5%) | 28/55 (50.9%) | 89/163 (54.6%) | 0.50 |
| LCX/OM/ramus | 25/108 (23.1%) | 10/55 (18.2%) | 35/163 (21.5%) | 0.47 |
| RCA/RPDA/RPL | 25/108 (23.1%) | 18/55 (32.7%) | 43/163 (26.4%) | 0.19 |
| Left Main | 0/108 (0.0%) | 0/55 (0.0%) | 0/163 (0.0%) | NA |
| Bypass vessel | 0/108 (0.0%) | 0/55 (0.0%) | 0/163 (0.0%) | NA |
| Diameter stenosis, % | 86.5±8.5 | 84.9±9.4 | 86.0±8.8 | 0.27 |
| Reference vessel diameter, mm | 2.92±0.44 | 3.00±0.42 | 2.95±0.43 | 0.21 |

Data are n/N (%) or mean±standard deviation. *Diameter stenosis >50% by quantitative coronary angiography. NSTEMI = non-ST elevation myocardial infarction; LAD = left anterior descending; LCX = left circumflex; OM = obtuse marginal; RCA = right coronary artery; RPDA = right posterior descending artery; RPL = right postero-lateral.

**Supplemental Table 4. Baseline Characteristics According to Site Recruitment Volume**

|  | **Low Recruiting Sites (<20 Patients)**  **n=388** | **High Recruiting Sites (≥20 Patients)**  **n=1241** | **Overall**  **N=1629** | **P-value** |
| --- | --- | --- | --- | --- |
| Age, years | 64.7±10.3 | 64.2±9.9 | 64.3±10.0 | 0.44 |
| Male sex | 293/388 (75.5%) | 929/1240 (74.9%) | 1222/1628 (75.1%) | 0.81 |
| Body mass index, kg/m^2^ | 29.0±6.1 | 29.2±5.6 | 29.2±5.7 | 0.41 |
| Hyperlipidemia | 322/388 (83.0%) | 928/1240 (74.8%) | 1250/1628 (76.8%) | 0.0009 |
| Hypertension | 319/388 (82.2%) | 866/1240 (69.8%) | 1185/1628 (72.8%) | <0.0001 |
| Diabetes | 141/388 (36.3%) | 353/1240 (28.5%) | 494/1628 (30.3%) | 0.003 |
| Current smoking | 74/388 (19.1%) | 287/1240 (23.1%) | 361/1628 (22.2%) | 0.09 |
| Prior myocardial infarction | 77/388 (19.8%) | 213/1240 (17.2%) | 290/1628 (17.8%) | 0.23 |
| Prior percutaneous coronary intervention | 134/388 (34.5%) | 336/1240 (27.1%) | 470/1628 (28.9%) | 0.005 |
| Target vessel percutaneous coronary intervention | 22/134 (16.4%) | 67/336 (19.9%) | 89/470 (18.9%) | 0.38 |
| Prior coronary artery bypass grafting | 14/388 (3.6%) | 62/1240 (5.0%) | 76/1628 (4.7%) | 0.26 |
| Prior stroke | 13/388 (3.4%) | 52/1240 (4.2%) | 65/1628 (4.0%) | 0.46 |
| History of congestive heart failure | 23/388 (5.9%) | 51/1240 (4.1%) | 74/1628 (4.5%) | 0.13 |
| Atrial fibrillation | 4/388 (1.0%) | 31/1240 (2.5%) | 35/1628 (2.1%) | 0.08 |
| Carotid disease | 26/387 (6.7%) | 45/1240 (3.6%) | 71/1627 (4.4%) | 0.009 |
| Peripheral artery disease | 20/388 (5.2%) | 64/1240 (5.2%) | 84/1628 (5.2%) | 1.00 |
| Ejection fraction, % | 58.3±8.5 | 57.3±9.2 | 57.6±9.0 | 0.031 |
| Clinical presentation |  |  |  |  |
| Stable angina | 207/388 (53.4%) | 598/1240 (48.2%) | 805/1628 (49.4%) | 0.08 |
| Unstable angina | 17.3% (67/388) | 265/1240 (21.4%) | 332/1628 (20.4%) | 0.08 |
| Braunwald classification |  |  |  |  |
| I | 21/67 (31.3%) | 85/265 (32.1%) | 106/332 (31.9%) | 0.91 |
| II | 11/67 (16.4%) | 86/265 (32.5%) | 97/332 (29.2%) | 0.010 |
| III | 35/67 (52.2%) | 94/265 (35.5%) | 129/332 (38.9%) | 0.012 |
| Silent ischemia | 46/388 (11.9%) | 104/1240 (8.4%) | 150/1628 (9.2%) | 0.039 |
| NSTEMI | 68/388 (17.5%) | 273/1240 (22.0%) | 341/1628 (20.9%) | 0.058 |
| Number of diseased vessels* |  |  |  |  |
| 1 | 292/388 (75.3%) | 871/1240 (70.2%) | 1163/1628 (71.4%) | 0.056 |
| 2 | 73/388 (18.8%) | 268/1240 (21.6%) | 341/1628 (20.9%) | 0.24 |
| 3 | 18/388 (4.6%) | 89/1240 (7.2%) | 107/1628 (6.6%) | 0.08 |
| ≥4 | 5/388 (1.3%) | 12/1240 (1.0%) | 17/1628 (1.0%) | 0.57 * |
| Diseased vessels |  |  |  |  |
| LAD/Diagonal | 217/388 (55.9%) | 734/1240 (59.2%) | 951/1628 (58.4%) | 0.25 |
| LCX/OM/ramus | 135/388 (34.8%) | 448/1240 (36.1%) | 583/1628 (35.8%) | 0.63 |
| RCA/RPDA/RPL | 153/388 (39.4%) | 521/1240 (42.0%) | 674/1628 (41.4%) | 0.37 |
| Left Main | 5/388 (1.3%) | 12/1240 (1.0%) | 17/1628 (1.0%) | 0.57 * |
| Bypass vessel | 3/388 (0.8%) | 9/1240 (0.7%) | 12/1628 (0.7%) | 1.00 * |
| Diameter stenosis, % | 85.0±9.8 | 84.0±9.9 | 84.3±9.9 | 0.11 |
| Reference vessel diameter, mm | 2.96±0.45 | 2.91±0.42 | 2.92±0.43 | 0.11 |
| P2Y12 loading dose administered | 156/259 (60.2%) | 582/827 (70.4%) | 738/1086 (68.0%) | 0.002 |
| Clopidogrel | 82/259 (31.7%) | 325/827 (39.3%) | 407/1086 (37.5%) | 0.027 |
| Ticlopidine | 1/259 (0.4%) | 3/827 (0.4%) | 4/1086 (0.4%) | 1.00 |
| Prasugrel | 7/259 (2.7%) | 31/827 (3.7%) | 38/1086 (3.5%) | 0.42 |
| Ticagrelor | 66/259 (25.5%) | 223/827 (27.0%) | 289/1086 (26.6%) | 0.64 |

Data are n/N (%) or mean±standard deviation. *Diameter stenosis >50% by quantitative coronary angiography. NSTEMI = non-ST elevation myocardial infarction; LAD = left anterior descending; LCX = left circumflex; OM = obtuse marginal; RCA = right coronary artery; RPDA = right posterior descending artery; RPL = right postero-lateral.

**Supplemental Table 5. Procedural Characteristics According to Site Recruitment Volume**

|  | **Low Recruiting Sites (<20 Patients)**  **n=388** | **High Recruiting Sites (≥20 Patients)**  **n=1241** | **Overall**  **N=1629** | **P-value** |
| --- | --- | --- | --- | --- |
| Vascular access site |  |  |  |  |
| Radial | 296/388 (76.3%) | 1012/1240 (81.6%) | 1308/1628 (80.3%) | 0.021 |
| Femoral | 87/388 (22.4%) | 225/1240 (18.1%) | 312/1628 (19.2%) | 0.06 |
| Brachial | 5/388 (1.3%) | 3/1240 (0.2%) | 8/1628 (0.5%) | 0.022* |
| Number of treated vessels |  |  |  |  |
| 1 | 348/388 (89.7%) | 1084/1240 (87.4%) | 1432/1628 (88.0%) | 0.23 |
| 2 | 39/388 (10.1%) | 155/1240 (12.5%) | 194/1628 (11.9%) | 0.19 |
| 3 | 1/388 (0.3%) | 1/1240 (0.1%) | 2/1628 (0.1%) | 0.42* |
| Target vessel(s) | N=306 | N=998 | N=1304 |  |
| LAD/diagonal | 146/306 (47.7%) | 447/998 (44.8%) | 593/1304 (45.5%) | 0.37 |
| LCX/OM/ramus | 70/306 (22.9%) | 268/998 (26.9%) | 338/1304 (25.9%) | 0.16 |
| RCA/RPDA/RPL | 89/306 (29.1%) | 283/998 (28.4%) | 372/1304 (28.5%) | 0.81 |
| Left main | 1/306 (0.3%) | 0/998 (0.0%) | 1/1304 (0.1%) | 0.23 |
| No of target lesions | 1.1±0.3 | 1.1±0.3 | 1.1±0.3 | 0.24 |
| Pre-dilatation of target vessel | 263/306 (85.9%) | 759/998 (76.1%) | 1022/1304 (78.4%) | 0.0002 |
| No of stent(s) implanted per target lesion | 1.2±0.5 | 1.2±0.5 | 1.2±0.5 | 0.59 |
| Maximal stent diameter, mm | 3.0±0.4 | 3.0±0.4 | 3.0±0.4 | 0.20 |
| Total stent length, mm | 28.4±15.7 | 26.5±13.8 | 26.9±14.3 | 0.020 |
| Stent post-dilated | 219/302 (72.5%) | 459/994 (46.2%) | 678/1296 (52.3%) | <0.0001 |
| Use of fractional flow reserve | 32/306 (10.5%) | 66/998 (6.6%) | 98/1304 (7.5%) | 0.026 |
| Use of intravascular ultrasound | 100/306 (32.7%) | 100/998 (10.0%) | 200/1304 (15.3%) | <0.0001 |
| Procedure duration, min | 51.4±30.6 | 49.1±112.9 | 49.6±99.6 | 0.001 |
| Procedural antiplatelet medications |  |  |  |  |
| Glycoprotein IIb/IIIa inhibitors | 18/388 (4.6%) | 66/1240 (5.3%) | 84/1628 (5.2%) | 0.60 |
| Aspirin loading dose administered | 184/388 (47.4%) | 581/1240 (46.9%) | 765/1628 (47.0%) | 0.84 |
| Cangrelor | 16/388 (4.1%) | 3/1240 (0.2%) | 19/1628 (1.2%) | <0.0001 |
| Lesion success* | 445/ 448 (99.3%) | 1479/1483 (99.7%) | 1924/1931 (99.6%) | 0.23 |
| Device success† | 428/437 (97.9%) | 1424/1457 (97.7%) | 1852/1894 (97.8%) | 0.87 |
| Procedure success‡ | 367/ 379 (96.8%) | 1189/1232 (96.5%) | 1556/1611 (96.6%) | 0.76 |

Data are n/N (%) or mean ± standard deviation. *Lesion success: Attainment of final in-stent residual diameter stenosis of <30% (by QCA), using any percutaneous method. †Device success: Attainment of final in-stent residual diameter stenosis of <30% of the target lesion (by QCA) using the assigned device. Analysis includes all target lesions in the denominator, whether an attempt to implant the assigned device was made. Lesions treated with multiple stents in which one stent is an assigned device and >1 is not the assigned device are considered as device failures. ‡Procedure success: Lesion success, without the occurrence of in-hospital MACE events, defined as a composite of all-cause death, myocardial infarction, and target vessel revascularization during the hospital stay. LAD = left anterior descending; LCX = left circumflex; OM = obtuse marginal; RCA = right coronary artery; RPDA = right posterior descending artery; RPL = right postero-lateral.
